# Electrical Impedance Analysis for Lung Cancer: A Prospective, Multicenter, Blind Validation Study

**DOI:** 10.3389/fonc.2022.900110

**Published:** 2022-07-20

**Authors:** Dawei Yang, Chuanjia Gu, Ye Gu, Xiaodong Zhang, Di Ge, Yong Zhang, Ningfang Wang, Xiaoxuan Zheng, Hao Wang, Li Yang, Saihua Chen, Pengfei Xie, Deng Chen, Jinming Yu, Jiayuan Sun, Chunxue Bai

**Affiliations:** ^1^ Department of Pulmonary Medicine and Critical Care Medicine, Zhongshan Hospital, Fudan University, Shanghai, China; ^2^ Shanghai Respiratory Research Institution, Shanghai, China; ^3^ Chinese Alliance Against Lung Cancer, Shanghai, China; ^4^ Shanghai Engineer & Technology Research Center of Internet of Things for Respiratory Medicine, Shanghai, China; ^5^ Department of Respiratory Endoscopy, Shanghai Chest Hospital, Shanghai Jiao Tong University, Shanghai, China; ^6^ Department of Respiratory and Critical Care Medicine, Shanghai Chest Hospital, Shanghai Jiao Tong University, Shanghai, China; ^7^ Department of Thoracic Surgery, Shanghai Pulmonary Hospital, Tongji University, Shanghai, China; ^8^ Department of Pulmonary Medicine, Nantong Tumor Hospital, Nantong, China; ^9^ Department of Thoracic Surgery, Zhongshan Hospital, Fudan University, Shanghai, China; ^10^ Key Laboratory of Public Health Safety, Ministry of Education, School of Public Health, Fudan University, Shanghai, China; ^11^ Shanghai Engineering Research Center of Respiratory Endoscopy, Shanghai, China

**Keywords:** electrical impedance, lung cancer, pulmonary nodules, diagnosis, prospective

## Abstract

**Hypothesis:**

Patients with cancer have different impedances or conductances than patients with benign normal tissue; thus, we can apply electrical impedance analysis (EIA) to identify patients with cancer.

**Method:**

To evaluate EIA’s efficacy and safety profile in diagnosing pulmonary lesions, we conducted a prospective, multicenter study among patients with pulmonary lesions recruited from 4 clinical centers (Zhongshan Hospital Ethics Committee, Approval No. 2015-16R and 2017-035(3). They underwent EIA to obtain an Algorithm Composite Score or ‘Prolung Index,’ PI. The classification threshold of 29 was first tested in an analytical validation set of 144 patients and independently validated in a clinical validation set of 418 patients. The subject’s final diagnosis depended on histology and a 2-year follow-up.

**Results:**

In total, 418 patients completed the entire protocol for clinical validation, with 186 true positives, 145 true negatives, 52 false positives, and 35 false negatives. The sensitivity, specificity, and diagnostic yield were 84% (95% CI 79.3%-89.0%), 74% (95% CI 67.4%-79.8%), and 79% (95%CI 75.3%-83.1%), respectively, and did not differ according to age, sex, smoking history, body mass index, or lesion types. The sensitivity of small lesions was comparable to that of large lesions (*p* = 0.13). Four hundred eighty-four patients who underwent the analysis received a safety evaluation. No adverse events were considered to be related to the test.

**Conclusion:**

Electrical impedance analysis is a safe and efficient tool for risk stratification of pulmonary lesions, especially for patients with a suspicious lung lesion.

## Highlights

- What is the key question?
Could we apply electrical impedance analysis (EIA) to identify patients with cancer?
- What is the bottom line?
EIA is a safe and efficient tool for risk stratification of pulmonary lesions, especially for patients presenting with a suspicious lung lesion.
- Why read on?
As a non-invasive test, EIA can be adjunctively incorporated with CT screening to both avoid overdiagnosis and missed diagnosis.

## Introduction

Lung cancer has become the most common incident cancer and the leading cause of cancer death in China (1). Low-dose computed tomography (LDCT) screening has reduced the mortality in high-risk populations (2, 3), but since LDCT involves the use of radiation, asymptomatic people are reluctant to undergo routine screening. Consequently, there is an unmet need for a non-invasive, radiation-free, and easy-to-use diagnostic tool for lung cancer risk stratification. Electrical impedance analysis (EIA) can be used to obtain impedance information related to human physiological and pathological conditions. In EIA, an electrode probe is placed on the body, and a small amount of current is passed through the body, allowing analysis of its impedance. The application scope of EIA includes monitoring pulmonary function, constructing functional brain imaging, evaluating body composition and nutrition status, and identifying tumors (4).

Bioimpedance has been valuable in detecting various cancers, including skin, thyroid, liver, cervix, and breast cancers (5–12), with breast cancer being the most extensively studied bioimpedance technology (9–12). It is well known that cancerous tissue has distinctly different electrical properties from non-cancerous tissue (13). This has been attributed to the high water and sodium content within cancerous tissues, altered membrane composition, the nucleus-to-cytoplasm ratio, and cellular composition and density (14–16). To construct an impedance analytical system for lung cancer detection, Kimura et al. (17) inserted an electric probe into the pulmonary mass during thoracotomy among 53 patients (17). They diagnosed 9 patients with intrathoracic lesions using the analytical system confirmed by a needle biopsy, resulting in no false negatives and only one false positive (17). Transcutaneous measurement of dermal impedance has been developed as an indication of internal organ pathologies, which was confirmed to be helpful in the diagnosis of lung cancer (18, 19). Current devices on the market to characterize biopsied tissue utilize what is generally referred to as Electrical Impedance Spectroscopy (EIS). Additional devices that monitor or image the various electrical properties of tissues are typically referred to as using Electrical Impedance Tomography (EIT). While the ProLung device shares some common aspects of these other technologies, it is unique in that the device does not measure or image the tumor nodule directly, but rather measures the systemic changes (bulk resistive changes to the interstitial fluids within the extracellular matrix and lymph system due to the presence of cancer in the body), which are significant and measurable due to the presence of cancer in the body (14, 20–26). Therefore, its accuracy is less affected by the size of the lesion, unlike traditional imaging technologies such as CT and PET scans.

In our previous study, we developed an EIA approach using 31 bilateral points on the skin surface (27). We achieved 89.7% sensitivity and 91.7% specificity in distinguishing between a cohort of lung cancer patients and healthy volunteers. This study optimized the detection sites to 20 skin surface points and updated the algorithm to compute an Algorithm Composite Score combining the impedance results from all detection sites. Based on our previous study, we enrolled more participants in this prospective, multicenter, blind validation study aiming to confirm EIA’s efficacy and safety profile in lung cancer diagnosis.

## Materials and Methods

### Participants

From June 2015 to August 2019, we recruited consecutive patients with pulmonary lesions suspected to be lung cancer from 4 clinical centers in China: Zhongshan Hospital Fudan University, Shanghai Chest Hospital, Shanghai Pulmonary Hospital, and Nantong Tumor Hospital. Patients recruited between June 2015 and June 2016 were enrolled in an analytical validation set for testing of the previously reported method and threshold. Patients recruited between October 2017 and August 2019 were enrolled in a clinical validation set for independent validation.

Inclusion criteria were as follows (1). Age 18–80 years (2). Presence of a pulmonary lesion 4–50 mm in diameter (3). Provision of a CT/PET (positron emission tomography) within 30 days. Exclusion criteria were as follows (1). Benign tumors of the central nervous system or other known malignancies, except for non-melanoma skin cancer, during the past five years (2). Confounding factors affecting thoracic impedance, including apparent pulmonary inflammation, tuberculosis, pleural effusion, thoracic anatomy abnormality, thoracic interventional therapy, implanted electric devices, dermatosis, and thoracic radiation or chemotherapy within the last 30 days (3). Other factors affecting thoracic anatomy and conductivity properties, such as strenuous exercise within the last 24 hours (4). Pregnancy or lactation (5). Presence of an unusually low conductivity, such as an Algorithm Composite Score of <20 when measured between the two hands following a 5-minute dwell time.

All patients provided written informed consent and agreed to undergo histological diagnosis. The ethics committee approved the protocol at each hospital. The trial was registered at www.clinicaltrials.gov (NCT02726633). All procedures involving human participants were as per the Declaration of Helsinki.

### Procedure

**Figure 1:**
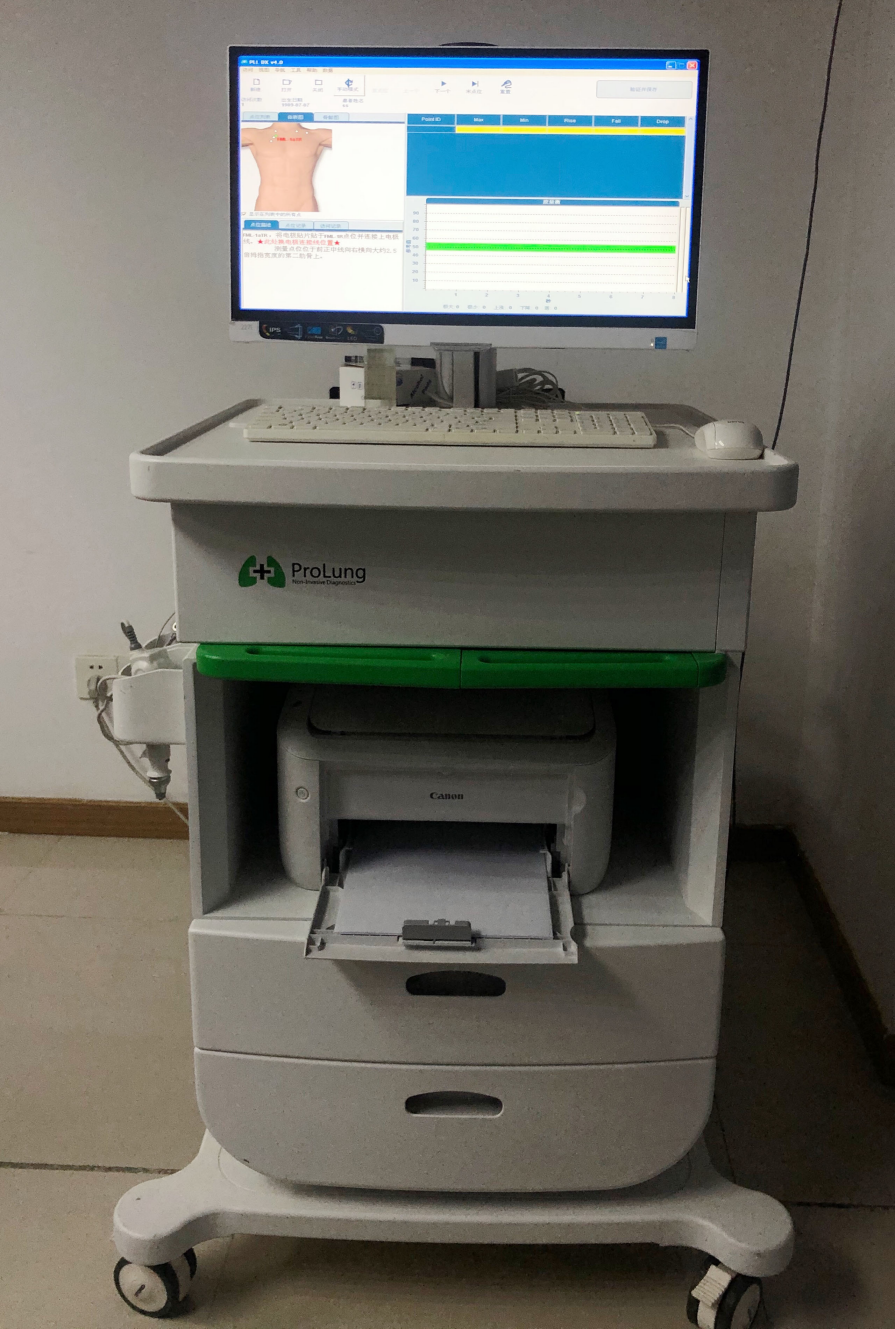
Electrical impedance analysis (EIA) platform.

The EIA (BSP-E2-1000-A, Prolung Biotech Wuxi Co, Wuxi, China) comprised a host computer, a reference electrode, and an impedance detector integrated with an electric probe (**Figure 1**). The electric probe passes a weak current (≤25 μA) through the body, forming a series circuit with the reference electrode. By sending a standard induction voltage to the interrogation location, the voltage difference across the body is detected, thus allowing the bioimpedance to be calculated. The operator measured 20 given sites on the surface of the skin. All data were delivered automatically to the host computer, and the algorithm generated an Algorithm Composite Score (or ‘Prolung Index,’ PI) for each point measured. The PI reveals the overall conductance property of the participants, which is the inverse of bioimpedance. Previous studies have shown that EIA can effectively distinguish malignancies from benign conditions; patients with Algorithm Composite Score ≥29 have a high risk for malignancy. Those with Algorithm Composite Score <29 are at low risk (27, 28). The same threshold was tested and validated in this study.

### Follow-Up

A final diagnosis was established by a comprehensive analysis of pathology and clinical follow-up. When the diagnosis was pathologically confirmed by surgery or biopsy, no follow-up was needed for newly discovered pulmonary lesions. A definitive result confirmed by biopsy should be a conclusive malignancy or a specific benign condition, such as a granuloma, fibrosis, or clear microbiological evidence.

In cases where a histological diagnosis was not performed, or the histology was indeterminate by biopsy, the patient underwent a 2-year clinical follow-up until further intervention was performed and a definitive histological diagnosis was established. Solid lesions that remained stable after two years were recorded as benign. Subsolid lesions were further discussed by a multi-disciplinary team consisting of two radiologists and one respiratory physician to determine whether they were benign. Histological examination was recommended when a follow-up CT showed morphological changes defined by the Clinical Practice Consensus Guidelines (29). Additional details on this method are provided in the online data supplement.

### Statistical Analysis

Statistical Analysis Software (SAS) (version 9.4) was used for statistical analyses. Descriptive statistics were recorded as frequency, percentage, range, and mean ± standard deviation. Categorical variables were compared using a chi-square test or Fisher’s exact probability test. All tests were performed bilaterally. A *p* value less than 0.05 was considered to be statistically significant.

## Results

### Study Cohorts

One hundred sixty-three participants with CT-detected pulmonary lesions were prospectively enrolled in the analytical validation set. Of these, 19 patients dropped out without a definitive diagnosis, resulting in 144 eligible patients for threshold testing. The baseline characteristics of the patients are shown in **Table 1**. Of the 144 cases, 60 had surgery, 68 had a nonsurgical bronchoscopic biopsy, and 16 had a nonsurgical fine-needle aspiration (FNA) biopsy. All nonsurgical patients were followed up for two years. The final diagnoses revealed 25 benign lesions and 119 lung cancers, including 20 squamous cell carcinomas, 88 adenocarcinomas, 3 other types of non-small-cell lung cancer, 4 small cell lung cancers, and 4 malignancies not otherwise specified.

**Table 1:** Baseline characteristics of the patients.

Characteristics	Analytical Validation Dataset (N=144)		Clinical Validation Dataset			
		Total	Center A	Center B	Center C	Center D
		(N=418)	(n=137)	(n=149)	(n=79)	(n=53)
Age, year						
Mean (SD)	60.2 (9.41)	57.8 (11.6)	57.3 (12.2)	59.0 (11.4)	53.4 (11.2)	62.3 (8.62)
Median [Min, Max]	61.0 [31.0, 79.0]	60 [20, 80]	60 [20, 80]	61 [32, 80]	56 [26, 73]	64 [45, 77]
Gender, n (%)						
Male	90 (62.5)	185 (44.3)	59 (43.1)	63 (42.3)	35 (44.3)	28 (52.8)
Female	54 (37.5)	233 (55.7)	78 (56.9)	86 (57.7)	44 (55.7)	25 (47.2)
BMI						
Mean (SD)	23.7 (3.20)	23.3 (3.27)	23.2 (3.03)	23.2 (3.50)	23.4 (2.88)	24.0 (3.74)
Median [Min, Max]	23.6 [15.4, 31.1]	23.1 [14.5, 37.9]	23.4 [16.9, 37.9]	22.8 [14.5, 31.2]	23.3 [18.0, 31.9]	23.1 [17.6, 32.0]
Smoke, n (%)						
No	101 (70.1)	305 (73.0)	101 (73.7)	106 (71.1)	63 (79.7)	35 (66.0)
Yes	43 (29.9)	113 (27.0)	36 (26.3)	43 (28.9)	16 (20.3)	18 (34.0)
Lesion Size, mm						
Mean (SD)	28.0 (13.1)	16.0 (10.8)	14.6 (10.2)	17.6 (10.6)	11.3 (8.96)	22.5 (11.6)
Median [Min, Max]	26.0 [4.90, 50.0]	13.0 [4.00, 50.0]	11.0 [4.00, 48.3]	15.0 [4.00, 46.0]	8.0 [4.00, 46.0]	21.0 [4.00, 50.0]
Lobe Location, n (%)						
RLL/LLL	56 (38.9)	117 (28.0)	33 (24.1)	42 (28.2)	16 (20.3)	26 (49.1)
RML	19 (13.2)	47 (11.2)	18 (13.1)	10 (6.71)	13 (16.5)	6 (11.3)
RUL/LUL	69 (47.9)	254 (60.8)	86 (62.8)	97 (65.1)	50 (63.3)	21 (39.6)
Lesion Type, n (%)						
MGGO	7 (4.9)	110 (26.3)	28 (20.4)	30 (20.1)	16 (20.3)	36 (67.9)
PGGO	21 (14.6)	146 (34.9)	55 (40.1)	40 (26.8)	45 (57.0)	6 (11.3)
Solid	111 (77.1)	161 (38.5)	54 (39.4)	79 (53.0)	18 (22.8)	10 (18.9)
Other	5 (3.5)	1 (0.2)	0 (0)	0 (0)	0 (0)	1 (1.9)
Final Diagnosis, n (%)						
Benign	25 (17.4)	197 (47.1)	72 (52.6)	62 (41.6)	54 (68.4)	9 (17.0)
Malignant	119 (82.6)	221 (52.9)	65 (47.4)	87 (58.4)	25 (31.6)	44 (83.0)
SQ	20 (13.9)	19 (4.5)	3 (2.2)	10 (6.7)	2 (2.5)	4 (7.5)
Ad	88 (61.1)	190 (45.5)	61 (44.5)	69 (46.3)	22 (27.8)	38 (71.7)
SCLC	4 (2.8)	4 (1.0)	1 (0.7)	1 (0.7)	0 (0)	2 (3.8)
NOS	4 (2.8)	6 (1.4)	0 (0)	6 (4.0)	0 (0)	0 (0)
Other malignancy	0 (0)	2 (0.5)	0 (0)	1 (0.7)	1 (1.3)	0 (0)
Diagnostic Method, n (%)						
Biopsy & follow-up	84 (58.3)	84 (20.1)	14 (10.2)	69 (46.3)	0 (0)	1 (1.9)
Surgery	60 (41.7)	193 (46.2)	76 (55.5)	36 (24.2)	31 (39.2)	50 (94.3)
Follow-up only		141 (33.7)	47 (34.3)	44 (29.5)	48 (60.8)	2 (3.8)

AD, adenocarcinoma; Center A**, **Zhongshan Hospital Fudan University; Center B**, **Shanghai Chest Hospital; Center C**,** Shanghai Pulmonary Hospital; Center D**,** Nantong Tumor Hospital; FNA, fine needle aspiration; LLL, left lower lobe; LUL, left upper lobe; MGGO, mixed ground-glass opacity; NOS, not otherwise specified; NSCLC, non-small cell lung cancer; Another malignancy refers to 1 non-small-cell lung cancer confirmed by pathology in Shanghai Chest Hospital and one combined adenocarcinoma and large cell neuroendocrine carcinoma in Shanghai Pulmonary Hospital; PGGO, pure ground-glass opacity; RLL, right lower lobe; RML, right middle lobe; RUL, right upper lobe; SD, standard deviation; SQ, squamous cell carcinoma; SCLC, small cell lung cancer.

**Figure 2:**
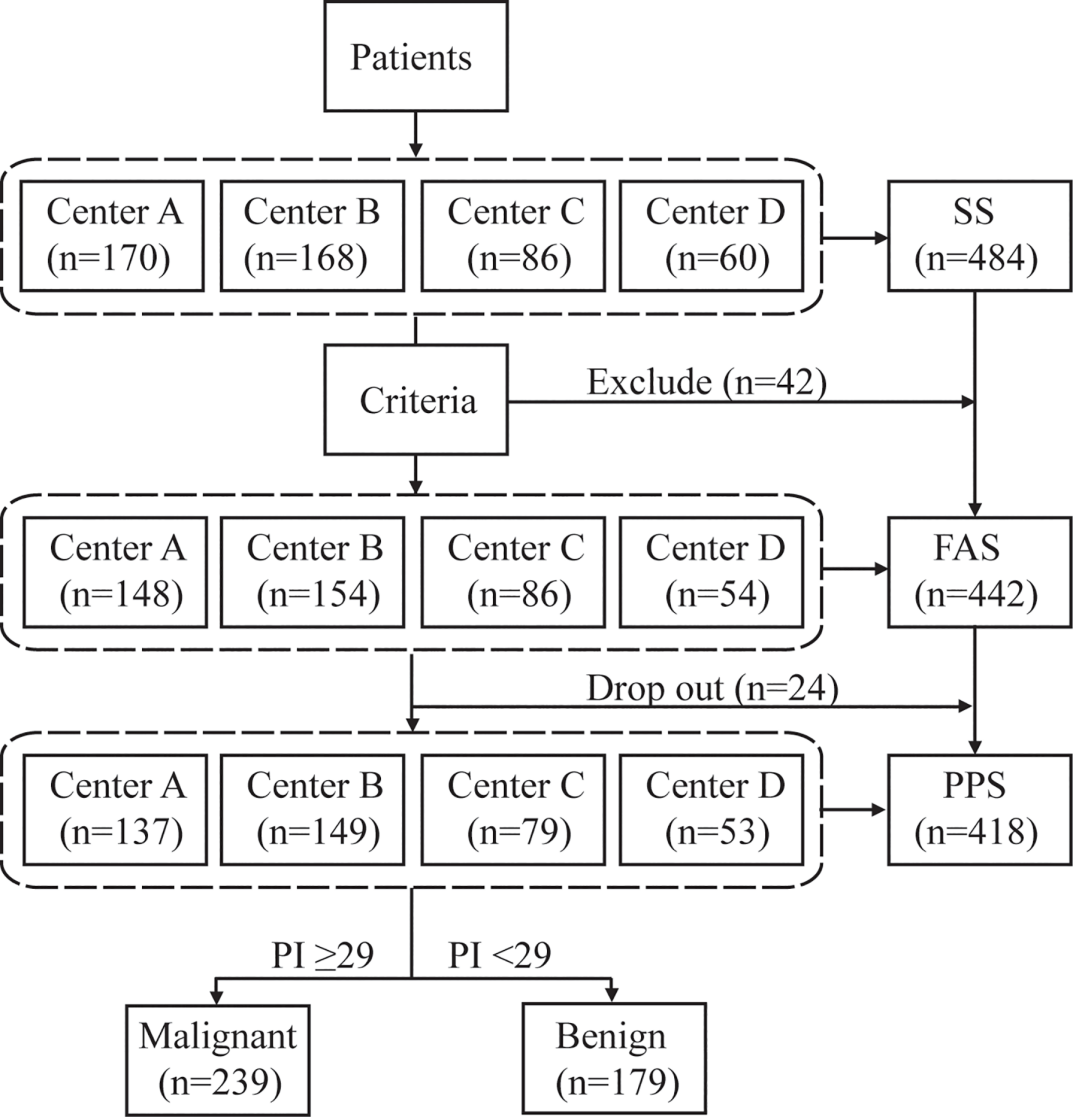
Study design flow chart. The safety dataset (SS) was composed of all subjects who underwent EIA and had at least one safety evaluation. The full analysis set (FAS) was made up of all eligible participants who underwent EIA. The per protocol set (PPS) consisted of eligible patients who completed the whole study and excluded severe violations of the protocol. Forty-two patients who were not suitable for the test were excluded, of whom 21 did not have eligible CT, 9 had Algorithm Composite Score < 20, 4 had pulmonary mass >50 mm, 3 had nodules <4 mm, 2 received thoracic intervention therapy, 2 had tuberculosis, 1 had an implanted steel plate in the thorax, one was aged >80 years, 1 had cancer history within five years. Twenty-four patients dropped out of the study, 23 due to the absence of compliance, and one died of cancer progression.

Four hundred eighty-four participants were prospectively enrolled in the study to validate the diagnostic performance of EIA (**Figure 2**). Of these, 42 patients did not meet the inclusion criteria and were excluded. Another 24 patients dropped out without a definitive diagnosis, resulting in 418 patients in the final validation set. The baseline characteristics of patients are shown in **Table 1**. Of the 418 cases in the clinical validation set, 183 had surgery, 94 had a nonsurgical bronchoscopic biopsy or FNA biopsy, and 141 had at least two years of clinical follow-up. The final diagnoses revealed 197 benign lesions and 221 malignancies, including 19 squamous cell carcinomas, 190 adenocarcinomas, 4 small cell lung cancers, and 6 malignancies not otherwise specified, as well as 1 non-small-cell lung cancer confirmed by pathology at Shanghai Chest Hospital and 1 combined adenocarcinoma and large cell neuroendocrine carcinoma at Shanghai Pulmonary Hospital.

### Diagnostic Efficacy and Ease of Operation of EIA

EIA was first performed in the analytical validation set. There were 98 true positives, 18 true negatives, 7 false positives, and 21 false negatives in the EIA analysis (**Table 2**). The overall sensitivity, specificity, positive predictive value, negative predictive value, and diagnostic yield were 82% (95% CI 75.5%-89.2%), 72% (95% CI 54.4%-89.6%), 93% (95% CI 88.6%-98.1%), 46% (95% CI 30.5%-61.8%), and 81% (95% CI 74.1%-87.0%), respectively. These results indicated that the Algorithm Composite Score threshold previously developed using a North American cohort was sufficiently applicable for Chinese cohorts.

**Table 2:** The diagnostic efficacy and ease of operation evaluation of electrical impedance analysis by different clinical centers.

Indicators	Analytical Validation Dataset (N=144)	Clinical Validation Dataset(N=418)	*P* Value	Clinical Validation Dataset				
				Center A(n=137)	Center B(n=149)	Center C(n=79)	Center D(n=53)	*P* value
ACC	0.81 (116/144)	0.79 (331/418)	0.817	0.77 (105/137)	0.78 (116/149)	0.82 (65/79)	0.85 (45/53)	0.530
Sens	0.82 (98/119)	0.84 (186/221)	0.783	0.85 (55/65)	0.83 (72/87)	0.88 (22/25)	0.84 (37/44)	0.944
Spec	0.72 (18/25)	0.74 (145/197)	1.000	0.69 (50/72)	0.71 (44/62)	0.80 (43/54)	0.89 (8/9)	0.394
PPV	0.93 (98/105)	0.78 (186/238)	0.001	0.71 (55/77)	0.80 (72/90)	0.67 (22/33)	0.97 (37/38)	0.005
NPV	0.46 (18/39)	0.81 (145/180)	<0.001	0.83 (50/60)	0.75 (44/59)	0.93 (43/46)	0.53 (8/15)	0.004
Kappa Value	0.445	0.580		0.535	0.541	0.623	0.577	
Easy Operation	100%	100%		100%	100%	100%	100%	

Center A**,** Zhongshan Hospital Fudan University; Center B**, **Zhongshan Hospital Fudan University; Center C**, **Shanghai Pulmonary Hospital; Center D**,** Nantong Tumor Hospital; ACC, accuracy; Sens, sensitivity; Spec, specificity; PPV, positive predictive value; NPV, negative predictive value.

When kappa value < 0, the consistency intensity is extremely poor; 0 ~ 0.20, faint; 0.21 ~ 0.40, weak; 0.41 ~ 0.60, moderate; 0.61 ~ 0.80, high; kappa > 0.81, extremely strong.

In the independent clinical validation set, there were 186 true positives, 145 true negatives, 52 false positives, and 35 false negatives in the EIA analysis (**Table 2**). The overall sensitivity, specificity, positive predictive value, negative predictive value, and diagnostic yield were 84% (95% CI 79.3%-89.0%), 74% (95% CI 67.4%-79.8%), 78% (95% CI 72.9%-83.4%), 81% (95% CI 74.8%-86.3%), and 79% (95% CI 75.3%-83.1%), respectively. The sensitivity, specificity, and diagnostic yield were statistically comparable for the four clinical centers. However, the positive (*p* = 0.005) and negative predictive values (*p* = 0.004) differed between the four clinical centers, potentially due to differences in their benign/malignant case ratios. The overall kappa value was 0.58 (95% CI 0.50–0.65), indicating moderate consistency.

### Influence of Clinical Variables on the Diagnostic Yield

The sensitivity, specificity, and diagnostic yield of EIA did not differ according to age, sex, body mass index, or patients’ smoking history (**Tables 3**, **4**). However, patients with malignant pulmonary lesions (85%) yielded a higher sensitivity than the specificity of patients with benign lesions (56%, *p* < 0.01, **Table 4**). For lesions <10 mm, 10–30 mm, and 30–50 mm, respectively, the diagnostic yield was 82% (95% CI 70.0%-94.1%), 77% (95% CI 70.5%-82.6%), and 84% (95% CI 73.8%-94.2%) (*p* = 0.45), with sensitivity of 88% (95% CI 76.2%-100.0%), 82% (95% CI 76.3%-88.4%), 90% (95% CI 81.2%-99.3%) (*p* = 0.35), and specificity of 69% (95% CI 44.1%-94.3%), 51% (95% CI 34.9%-67.0%), 56% (95% CI 23.1%-88.0%) (*p* = 0.55). The specificity decreased when evaluating subjects with large pulmonary lesions (>10 mm). The sensitivity, specificity and diagnostic yield did not vary among different lesions types, including solid lesions, pure GGOs, mixed GGOs, and patchy shadows.

**Table 3:** Diagnostic yield in the analytical validation dataset by different variables.

Variable	Diagnostic yield	*P-Value*	Sensitivity	*P-Value*	Specificity	*P-Value*
Age, year						
18~44	0.50 (4/8)	0.092	0.75 (3/4)	0.907	0.25 (1/4)	0.054
45~69	0.82 (91/111)		0.82 (75/92)		0.84 (16/19)	
≥70	0.84 (21/25)		0.87 (20/23)		0.50 (1/2)	
Gender						
Male	0.79 (71/90)	0.664	0.82 (59/72)	1.000	0.67 (12/18)	0.626
Female	0.83 (45/54)		0.83 (39/47)		0.86 (6/7)	
BMI						
<24	0.86 (66/77)	0.143	0.86 (57/66)	0.299	0.82 (9/11)	0.407
≥24	0.75 (50/67)		0.77 (41/53)		0.64 (9/14)	
Smoke						
No	0.84 (85/101)	0.149	0.85 (72/85)	0.425	0.81 (13/16)	0.205
Yes	0.72 (31/43)		0.76 (26/34)		0.56 (5/9)	
Lesion Size, mm						
<10	0.70 (7/10)	0.017	0.71 (5/7)	0.026	0.67 (2/3)	1.000
10~30	0.74 (56/76)		0.75 (47/63)		0.69 (9/13)	
30~50	0.91 (53/58)		0.94 (46/49)		0.78 (7/9)	
Lobe Location						
RLL/LLL	0.82 (46/56)	0.374	0.86 (37/43)	0.268	0.69 (9/13)	1.000
RML	0.68 (13/19)		0.69 (11/16)		0.67 (2/3)	
RUL/LUL	0.83 (57/69)		0.83 (50/60)		0.78 (7/9)	
Lesion Type						
MGGO	1 (7/7)	0.337	1 (5/5)	0.585	1 (2/2)	0.718
PGGO	0.76 (16/21)		0.76 (13/17)		0.75 (3/4)	
Solid	0.79 (88/111)		0.82 (77/94)		0.65 (11/17)	
Patchy shadows	1 (5/5)		1 (3/3)		1 (2/2)	
Final Diagnosis						
Benign	0.72 (18/25)	0.262			0.72 (18/25)	
Malignant	0.82 (98/119)		0.82 (98/119)			
Diagnostic Method						
Biopsy	0.83 (70/84)	0.434	0.87 (58/67)	0.260	0.71 (12/17)	1.000
Surgery	0.77 (46/60)		0.77 (40/52)		0.75 (6/8)	

AD, adenocarcinoma; Center A**, **Zhongshan Hospital Fudan University; Center B**, **Shanghai Chest Hospital; Center C**, **Shanghai Pulmonary Hospital; Center D**,** Nantong Tumor Hospital; FNA, fine needle aspiration; LLL, left lower lobe; LUL, left upper lobe; MGGO, mixed ground-glass opacity; NOS, not otherwise specified; NSCLC, non-small cell lung cancer; PGGO, pure ground-glass opacity; RLL, right lower lobe; RML, right middle lobe; RUL, right upper lobe; SD, standard deviation; SQ, squamous cell carcinoma; SCLC, small cell lung cancer.

**Table 4:** Diagnostic yield in the clinical validation dataset by different variables.

Variable	Diagnostic yield	*P-Value*	Sensitivity	*P-Value*	Specificity	*P-Value*
Age, year						
18-44	0.78 (29/37)	0.28	0.79 (22/28)	0.07	0.78 (7/9)	0.35
45-69	0.81 (156/193)		0.88 (134/152)		0.54 (22/41)	
≥70	0.70 (33/47)		0.75 (30/40)		0.43 (3/7)	
Gender						
Male	0.82 (106/130)	1.00	0.87 (91/105)	0.52	0.60 (15/25)	0.80
Female	0.81 (112/138)		0.83 (95/115)		0.74 (17/32)	
BMI						
<24	0.75 (123/163)	0.15	0.82 (105/128)	0.30	0.51 (18/35)	0.53
≥24	0.83 (95/114)		0.88 (81/92)		0.64 (14/22)	
Smoke						
No	0.78 (143/184)	0.68	0.83 (119/144)	0.38	0.60 (24/40)	0.54
Yes	0.81 (75/93)		0.88 (67/76)		0.47 (8/17)	
Lesion size, mm						
<10	0.82 (32/39)	0.45	0.88 (23/26)	0.35	0.69 (9/13)	0.55
10~30	0.77 (144/188)		0.82 (126/153)		0.51 (18/35)	
30~50	0.84 (42/50)		0.90 (37/41)		0.56 (5/9)	
Lobe location						
RLL/LLL	0.80 (83/104)	0.26	0.83 (66/80)	0.01	0.71 (17/24)	0.03
RML	0.65 (15/23)		0.61 (11/18)		0.80 (4/5)	
RUL/LUL	0.80 (120/150)		0.89 (109/122)		0.39 (11/28)	
Lesion type						
MGGO	0.79 (59/75)	0.15	0.81 (51/63)	0.03	0.67 (8/12)	0.66
PGGO	0.80 (83/104)		0.85 (73/86)		0.56 (10/18)	
Solid	0.66 (25/38)		0.73 (22/30)		0.38 (3/8)	
Patchy shadows	0.85 (51/60)		0.98 (40/41)		0.58 (11/19)	
Final diagnosis						
Benign	0.56 (32/57)	<0.01			0.56 (32/57)	
Malignant	0.85 (186/220)		0.85 (186/220)			
Diagnostic method						
Biopsy	0.75 (63/84)	0.40	0.85 (51/60)	1.00	0.50 (12/24)	0.60
Surgery	0.80 (155/193)		0.84 (135/160)		0.61 (20/33)	

AD, adenocarcinoma; Center A**, **Zhongshan Hospital Fudan University; Center B**,** Shanghai Chest Hospital; Center C**,** Shanghai Pulmonary Hospital; Center D**,** Nantong Tumor Hospital; FNA, fine needle aspiration; LLL, left lower lobe; LUL, left upper lobe; MGGO, mixed ground-glass opacity; NOS, not otherwise specified; NSCLC, non-small cell lung cancer; Another malignancy refers to 1 combined adenocarcinoma and large cell neuroendocrine carcinoma in Shanghai Pulmonary Hospital; PGGO, pure ground-glass opacity; RLL, right lower lobe; RML, right middle lobe; RUL, right upper lobe; SD, standard deviation; SQ, squamous cell carcinoma; SCLC, small cell lung cancer.

Because a 2-year clinical follow-up was insufficient to establish a definitive diagnosis for subsolid lesions, we tabulated the diagnostic results of EIA. We then compared them with the findings obtained from pathology and clinical follow-up (**Table 5**). Of the pure GGOs, 44.52% (65/146) were diagnosed by pathology, resulting in a sensitivity, specificity, and diagnostic yield of 78% (45/58, 95% CI 66.9%-88.3%), 57% (4/7, 95% CI 20.5%-93.8%), 75% (49/65, 95% CI 64.9%-85.9%), respectively. A total of 81 pure GGOs were established through a final diagnosis by clinical follow-up, resulting in a sensitivity, specificity, and diagnostic yield of 100% (1/1), 80% (64/80, 95% CI 71.2%-88.8%), and 80% (65/81, 95% CI 71.6%-88.9%), respectively. Of the mixed GGOs, 81% (89/110) were diagnosed by pathology, resulting in a sensitivity, specificity, and diagnostic yield of 84% (64/76, 95% CI 76.0%-92.4%), 54% (7/13, 95% CI 26.7%-80.9%), 80% (71/89, 95% CI 71.4%-88.1%), respectively. A total of 21 mixed GGOs were established through a final diagnosis by clinical follow-up, resulting in a sensitivity, specificity, and diagnostic yield of 100% (1/1), 75% (15/20, 95% CI 56.0%-94.0%), and 76% (16/21, 95% CI 58.0%-94.4%), respectively.

**Table 5:** Diagnostic result of Electrical impedance analysis (EIA) compared with pathology and follow-up in subsolid lesions.

Lesion type	EIA	Pathology		Follow-up	
		+	-	+	-
Pure GGO	+	45	3	1	16
	–	13	4	0	64
Mixed GGO	+	64	6	1	5
	–	12	7	0	15

### Complications

The safety evaluation was conducted on 484 patients who were evaluated by EIA. No patient discomfort related to the measurement procedure was reported during the course of, or within 24 hours of, the operation. However, one patient died of lung cancer progression during clinical follow-up.

## Discussion

In this prospective, multicenter study assessing the use of EIA as a diagnostic tool for Chinese patients with pulmonary lesions, EIA was shown to be capable of safely and accurately discriminating between malignant and benign lesions with high sensitivity, specificity, and diagnostic yield. This accuracy was seen to be unaffected by patient demographics and clinical characteristics such as age, sex, smoking history, body mass index, or lesion types. Of note, the sensitivity associated with small lesions was comparable to that for large lesions.

EIA has long been used for electrocardiographs and electroencephalograms. It has also been utilized in skin cancer identification, thyroid nodule differentiation, and breast cancer risk stratification and screening (30–34). Stojadinovic et al. conducted breast cancer screening among 1,103 young women using EIA and established a sensitivity of 50% and a specificity of 90% (32, 33). No large-scale clinical trials have been conducted to evaluate EIA’s utility in pulmonary lesion risk stratification.

The American National Lung Screening Trial (NLST) demonstrated a relative reduction in lung cancer mortality of 20% following implementation of screening using three annual low-dose CT scans compared with planar chest radiographs among a high-risk population (3). However, using the NLST data, Bach et al. (35) estimated that approximately one radiation-associated cancer death would result per 2,500 people screened (35). Unlike CT, EIA does not employ ionizing radiation and so would be expected to have a lower risk of screening associated malignancies. CT screening is a trade-off that benefits people with potential high risk (aged 55–74 years, smoking history ≥30 pack-years). But for the low-risk population, the harm may outweigh the benefits (35, 36).

Following CT screening, false negatives result in late diagnosis and poor prognosis. False-positive CT readings lead to psychological distress, more frequent follow-up exposure to ionizing radiation (full dose CTs and PET-CT scans), and potentially unnecessary and harmful invasive procedures. Individuals with indeterminate pulmonary nodules >8 mm are recommended to undergo diagnostic procedures, such as PET, nonsurgical biopsy, surgery, and CT surveillance (37). Although the non-invasive PET has a sensitivity of 72–94% for malignant lesions, it is not very effective in diagnosing small nodules (<8–10 mm), pure GGOs, and mixed GGOs with a solid component ≤8 mm (37, 38), for which EIA has better performance than PET. The diagnostic yield of EIA for pure GGOs (78%) was not inferior to that for solid nodules (80%), mixed GGOs (79%), and patchy shadows (100%; p = 0.37), as was its sensitivity (p = 0.39) and specificity (p = 0.32). Additionally, EIA has a very impressive sensitivity of 85% for nodules <10 mm, which allows it to identify lung cancer at a very early stage. Based upon combined results from EIA and CT, clinicians could recommend a relatively conservative or a more aggressive intervention according to the test results.

In addition, an easy-to-use risk stratification method for use after CT screening is needed to avoid repeated radiation exposure in low-risk populations. As EIA had a good sensitivity (84%) and a better specificity (74%) than CT, it could be sufficiently accurate for use as a risk stratification tool. The positive (78%) and negative (81%) predictive values suggest that EIA could be used as a valid “rule out” test while effectively capturing early cancers. Unlike CT screening, EIA provides a direct and immediate conclusion after the test without requiring further interpretation by experts. When an EIA test result suggests a high malignancy risk, physicians could then advise the patients to undergo the necessary CT examinations or more invasive biopsies. EIA could also be beneficial for large-scale lung cancer risk stratification initiatives, especially in less developed geographical areas with limited access to medical professionals and advanced healthcare facilities, for people who are prone to psychological distress due to suspected illness, and for patients who cannot afford an annual physical examination.

We evaluated multiple variables that might affect the diagnostic efficacy of EIA. Since many participants had multiple pulmonary lesions, we only focused on lesions suspected to be malignant while regarding other lesions as normal. The sensitivity, specificity, and diagnostic yield were not affected by the patients’ age, sex, or body mass index. This confirms that EIA can be generally used in a wide range of cases, especially when there is suspicion of lung cancer, as it was more sensitive for malignant lesions (84%) than benign lesions (74%; *p* = 0.01). For lesions <10 mm, 10–30 mm, and 30–50 mm, the sensitivity was not significantly different (85%, 82%, and 90%, respectively; *p* = 0.43). The specificity, however decreased significantly for lesions of these sizes (82%, 61%, and 50%, respectively; *p* = 0.004). With the high sensitivity and specificity in nodules smaller than 10 mm, EIA may be more effective in the risk stratification of small pulmonary lesions. At the same time, certain clinical considerations may be necessary for excluding false-positive cases when identifying large pulmonary lesions. Because of the low reported specificity of CT, patients with sub-centimeter nodules, which have lower cancer risk than larger lesions, may be required to undergo successive annual CT follow-ups. Using EIA risk stratification, patients with small nodules could obtain a cancer risk assessment immediately after CT detection to help decide the optimal frequency of subsequent CT follow-ups. In addition, EIA could be used as an adjunctive follow-up test so that patients only need to receive CT detection when EIA indicates an increased risk. The distinct yet complementary diagnostic capabilities of EIA compared to CT make it a powerful tool to supplement, rather than challenge, the comprehensive analysis provided by CT imaging.

The sensitivity of EIA for lesions in the right middle lobe (56%) was lower than bilateral upper lobes (90%) and bilateral lower lobes (82%; *p* = 0.002), which may be because there were very few subjects with malignant lesions located in the right middle lobe (18/418). A single false negative would result in a 6% drop in sensitivity. EIA could be utilized for various lesion types, including solid lesions, pure GGOs, mixed GGOs, and patchy shadows that are suspected to be malignant. Of note, most patients with pure GGOs require a long period of clinical follow-up. However, EIA offers a risk stratification with a single test, making it easier for physicians to decide whether the pure GGOs should undergo invasive intervention.

There were some limitations in this study. First, The EIA suggests an overall but not the individual pathological status of a particular lesion. For patients with multiple pulmonary lesions, it cannot indicate which one needs further intervention. Second, the study enrolled patients with single and multiple pulmonary lesions, making it challenging to eliminate confounding factors caused by lesions other than the target lesions when analyzing the factors that affected diagnostic efficacy. Third, other variables such as lesion depth and tumor stage of the patients should be evaluated to see whether they affect the diagnostic effectiveness in order to learn more about the scope of EIA application. Finally, follow-up was limited to two years, which resulted in the inability to receive conclusive diagnoses for some pure GGOs. However, a two-year follow-up is sufficient to prove whether the lesion is stable and has a low risk of progression in the short term. Additionally, providing a conclusive pathological diagnosis for begin lesions arose since they were diagnosed by clinical follow-up.

In conclusion, Electrical Impedance Analysis (EIA) is a sufficiently accurate diagnostic tool that effectively detects the overall pathological conditions of pulmonary lesions. As a non-invasive test, it is very safe and easy to use. It can be adjunctively incorporated with CT screening to both avoid overdiagnosis and missed diagnosis.

## Data Availability Statement

The datasets presented in this article are not readily available because of confidentiality issues. Requests to access the datasets should be directed to CB, bai.chunxue@zs-hospital.sh.cn.

## Ethics Statement

The studies involving human participants were reviewed and approved by Zhongshan Hospital Fudan University Ethics Committee, Approval No. 2015-16R and 2017-035(3). The patients/participants provided their written informed consent to participate in this study.

## Author Contributions

Conceptualization and design: DY, JS, CB. Data curation, analysis, and interpretation: DY, CG, YG, XZ (4th Author), DG, YZ, NW, XZ (8th Author), HW, LY, SC, PX. Manuscript drafting: DY, CG, DC, JS, CB. Manuscript revision: DY, JY, MG, JS, CB. All authors have approved the version to be published and agree to be accountable for all aspects of the work.

## Funding

National Natural Science Foundation of China (82170110), Shanghai Pujiang Program (20PJ1402400), Science and Technology Commission of Shanghai Municipality (20DZ2254400, 21DZ2200600, 20DZ2261200) and Shanghai Municipal Key Clinical Specialty(shslczdzk02201).

## Conflict of Interest

The authors declare that the research was conducted in the absence of any commercial or financial relationships that could be construed as a potential conflict of interest.

## Publisher’s Note

All claims expressed in this article are solely those of the authors and do not necessarily represent those of their affiliated organizations, or those of the publisher, the editors and the reviewers. Any product that may be evaluated in this article, or claim that may be made by its manufacturer, is not guaranteed or endorsed by the publisher.
